# Supplementary figures and images for: Holistic integration of omics data reveals the drivers that shape the ecology of microbial meat spoilage scenarios
Source: Front Microbiol. 2023 Oct 18;14:1286661. doi: 10.3389/fmicb.2023.1286661 (PMC10619683; doi:10.3389/fmicb.2023.1286661)

Day 7

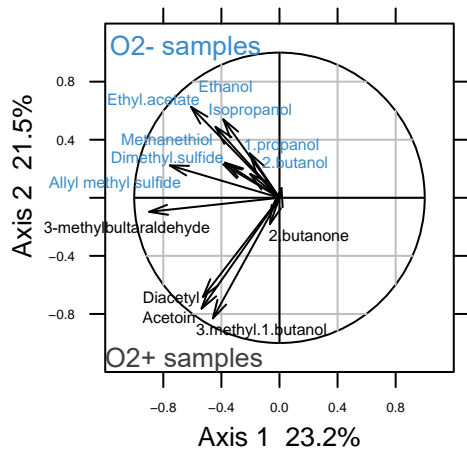

Day 15

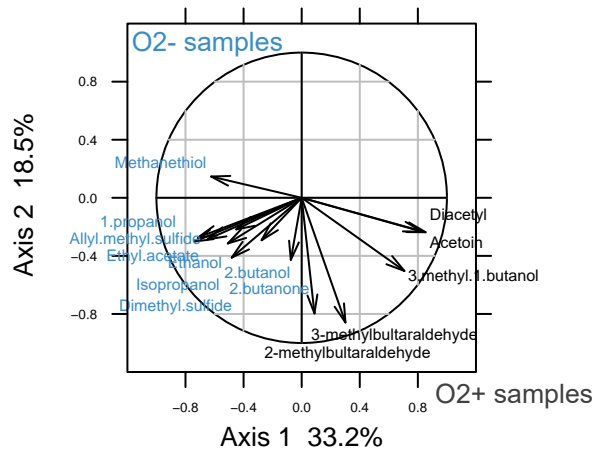

Day 22

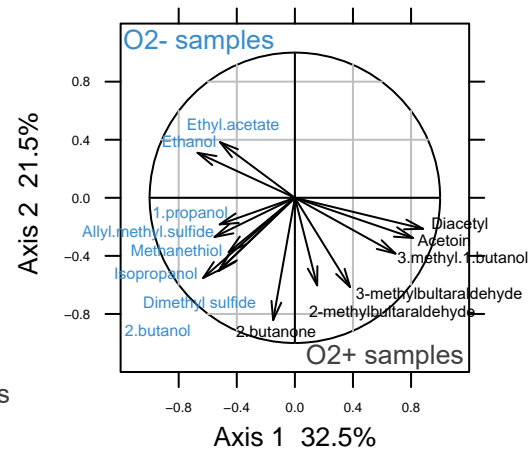

Poultry samples

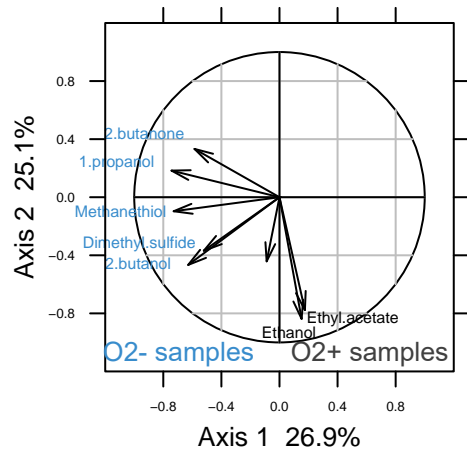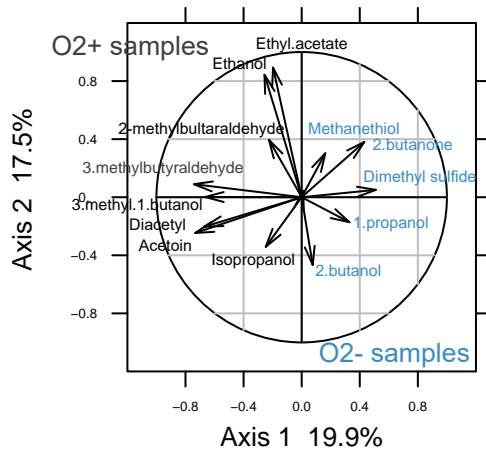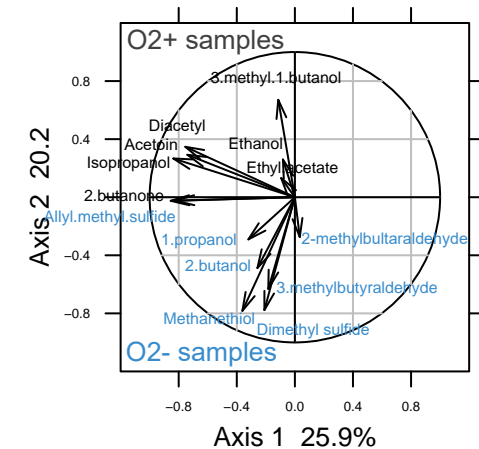

Pork samples

Supplement: Supplementary file 1 [file Data_Sheet_1.PDF]

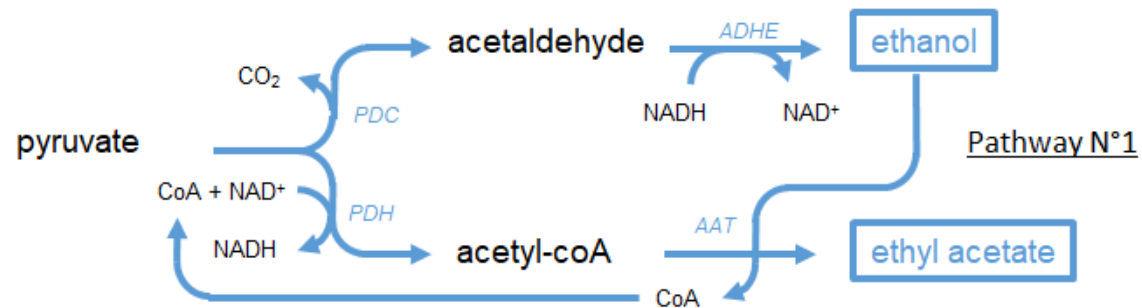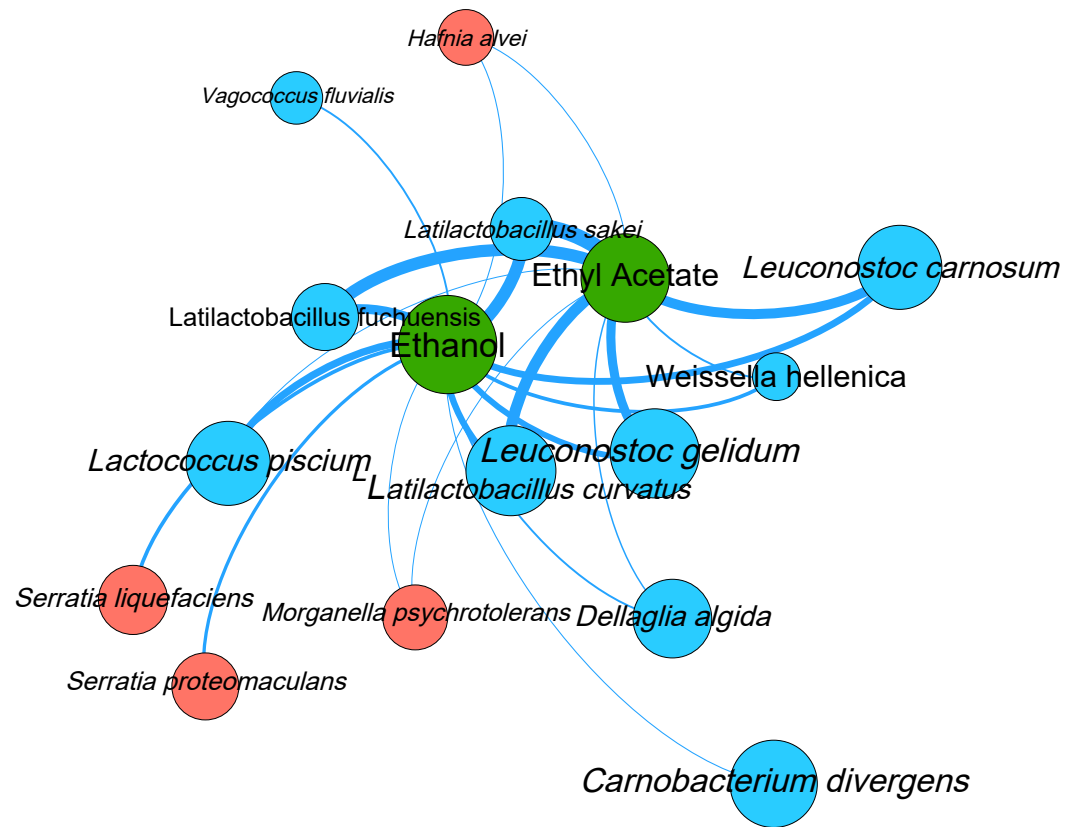

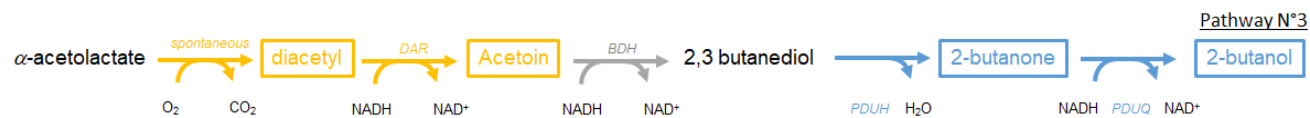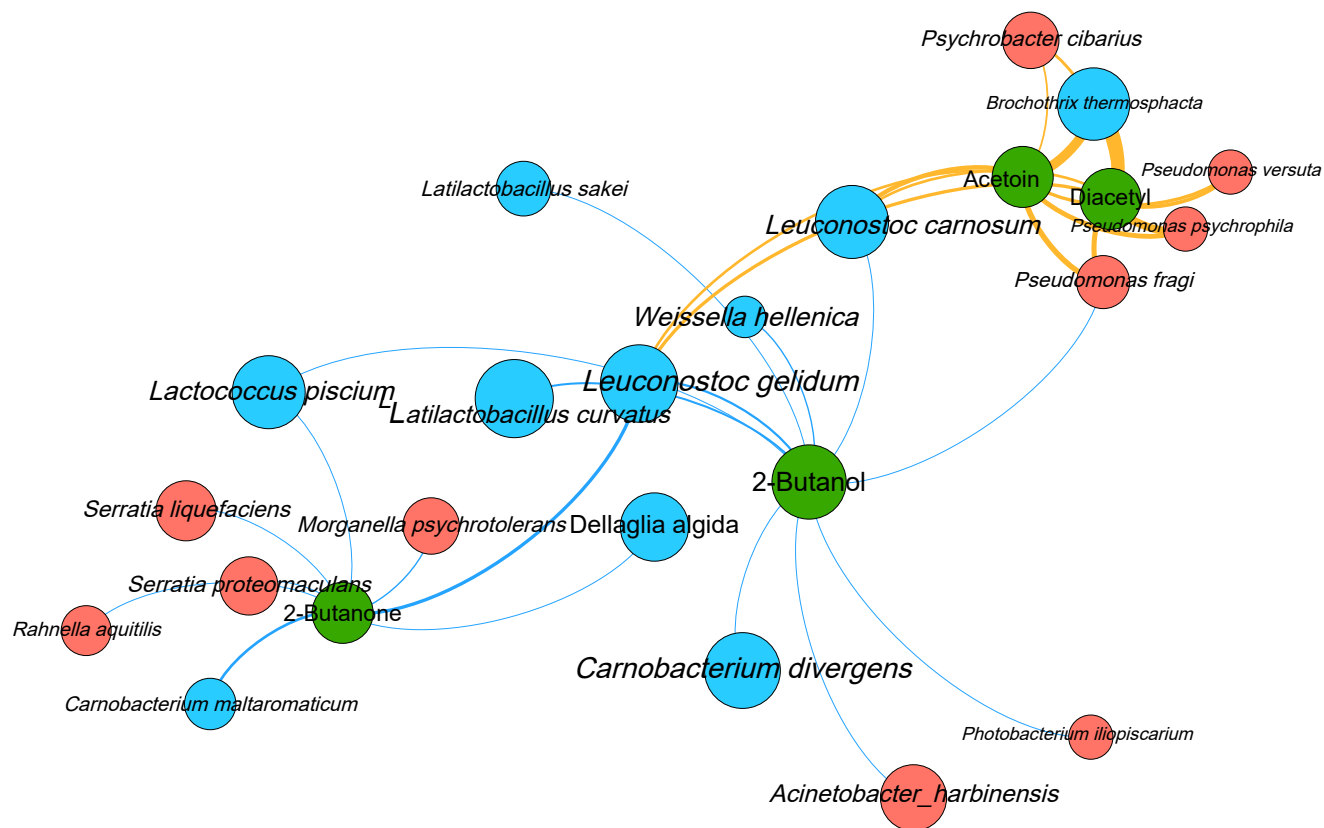

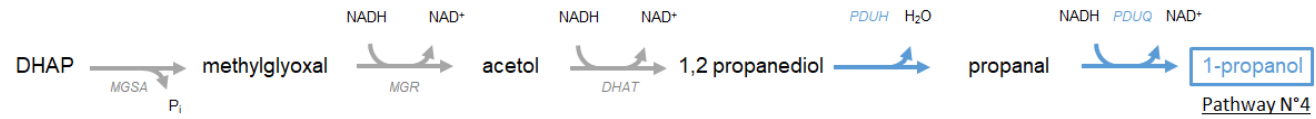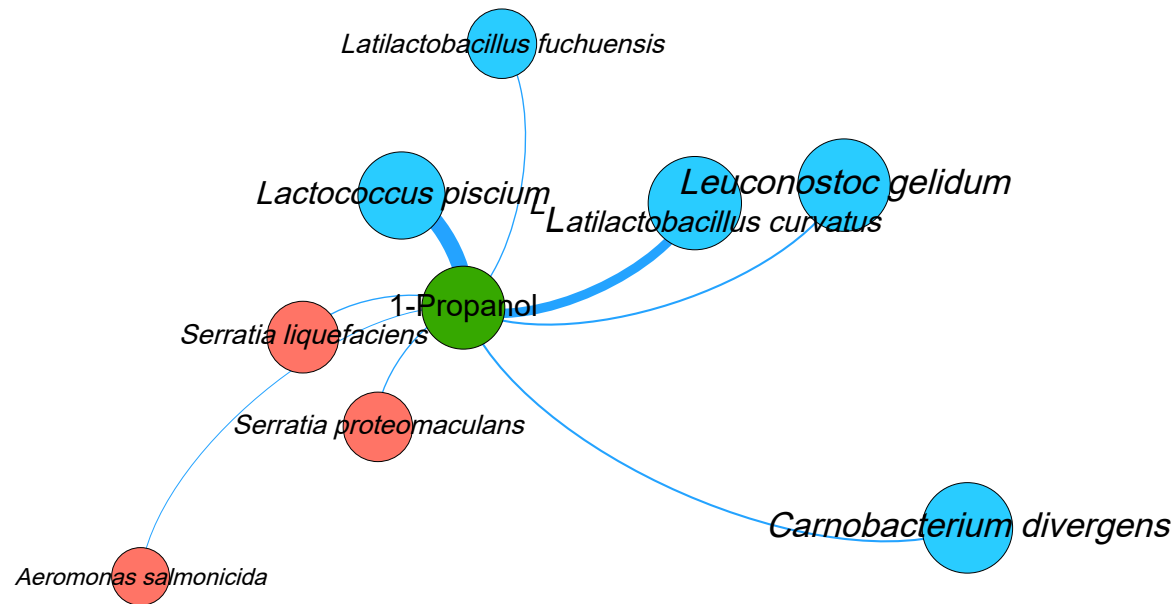

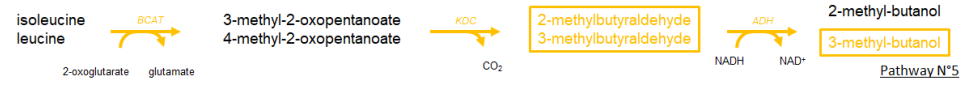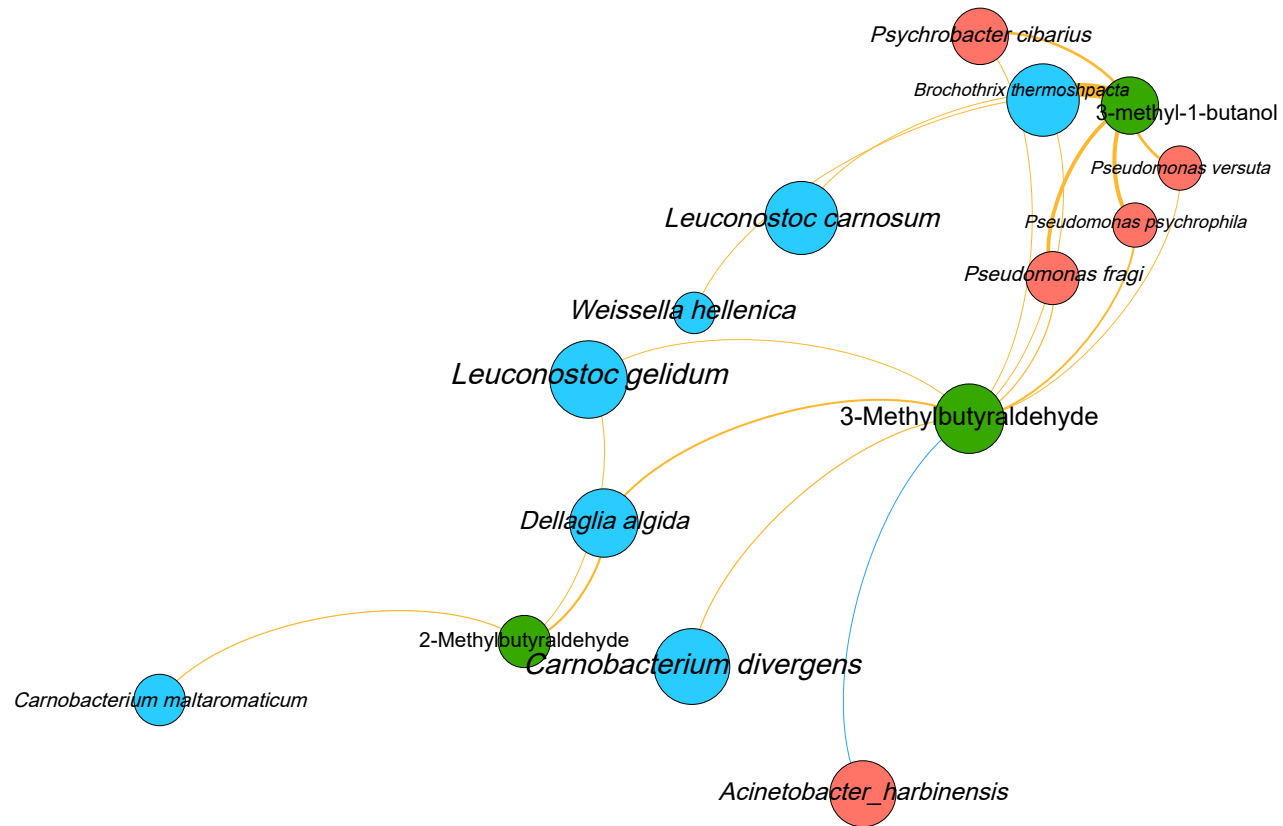

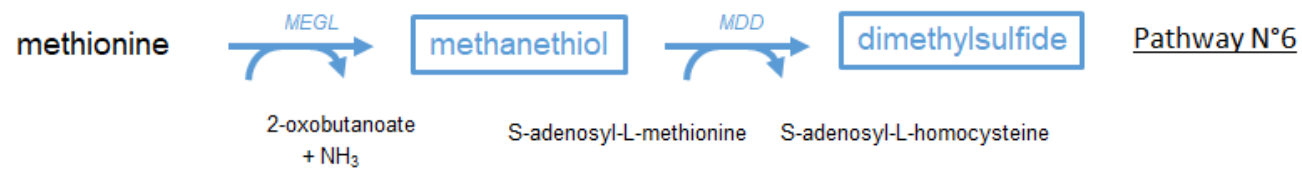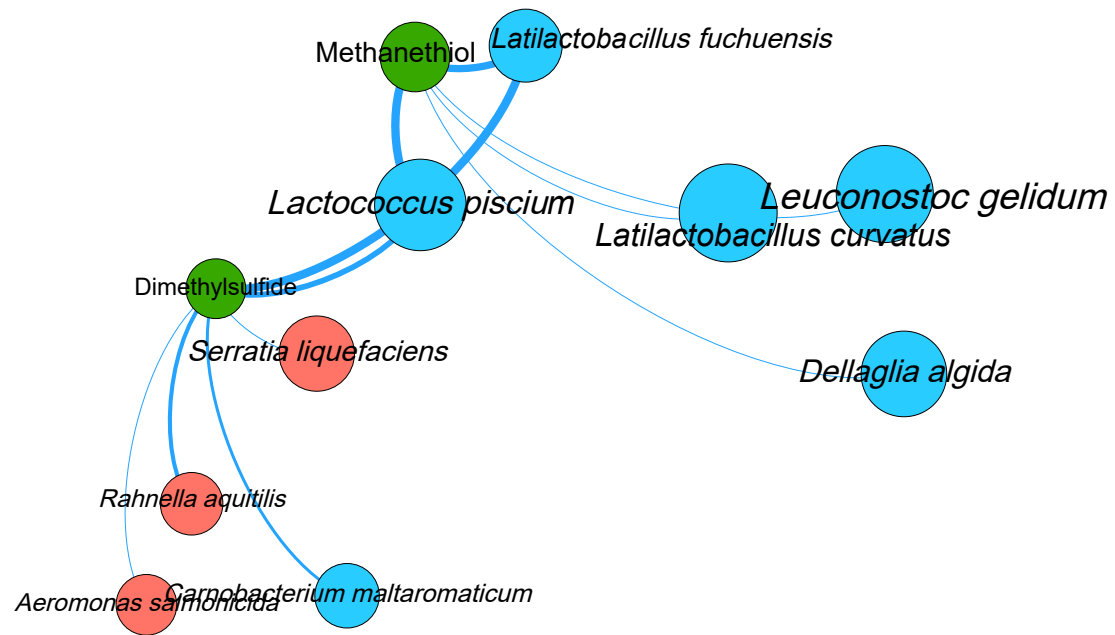

Supplement: Supplementary file 3 [file Data_Sheet_3.PDF]
