# Supplementary material for: Holistic integration of omics data reveals the drivers that shape the ecology of microbial meat spoilage scenarios
Source: Front Microbiol. 2023 Oct 18;14:1286661. doi: 10.3389/fmicb.2023.1286661 (PMC10619683; doi:10.3389/fmicb.2023.1286661)
Supplement: Supplementary file 2 [file Data_Sheet_2.PDF]

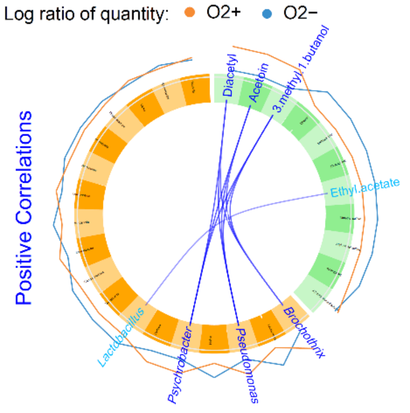

Correlation cut−off
r=0.7

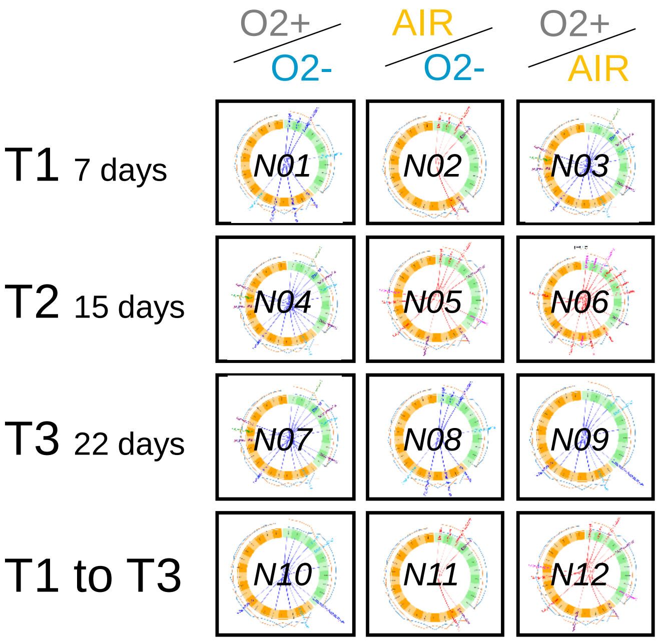

Samples	Time	O2+ / O2-	AIR / O2-	O2+ / AIR
Poultry samples	T1	22	7	0
	T2	19	10	0
	T3	26	10	7
	ALL	23	0	0
Pork samples	T1	1	0	0
	T2	4	0	0
	T3	10	2	0
	ALL	7	0	0
All samples	T1	7	2	0
	T2	25	4	0
	T3	12	16	0
	ALL	13	6	0

	O2+ / O2-	AIR / O2-	O2+ / AIR
T1	30	9	0
T2	48	14	0
T3	48	28	7
ALL	43	6	0

Positive interactions		Frequency	Weigtht	Modalities	
Source	Target	*nb* of hit	∑ of *r* values	Packaging	Time
Lactococcus piscium	Methanethiol	7	2.7	O2-	T2
Latilactobacillus curvatus	1-propanol	3	1.5	O2-	T3
Brochothrix thermosphacta	Acetoin	12	9.6	O2+	ALL
.../					

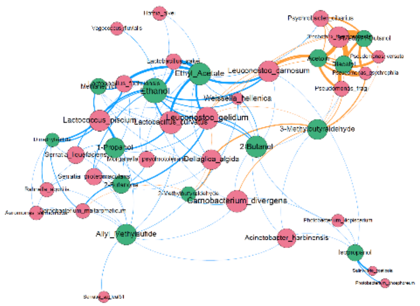

1. Design for mixDIABLO correlation analysis *N= 12 different correlations*

2. Number of positive correlations for different sample subsets = *233*

3. Summary of all correlations and their strengths to built ecological network
